# Supplementary material for: UK Dog Owners’ Pre-Acquisition Information- and Advice-Seeking: A Mixed Methods Study
Source: Animals (Basel). 2024 Mar 28;14(7):1033. doi: 10.3390/ani14071033 (PMC11010827; doi:10.3390/ani14071033)
Supplement: Supplementary file 1 [file animals-14-01033-s001.zip › animals-2916473-supplementary (1).pdf]

Supplementary Materials

Contents

A. "Choosing My Dog" survey questions relevant to pre-acquisition research.................................2
B. "Choosing My Dog" interview schedule 11
C. Participant recruitment 16
D. Coding of survey free text responses 17
E. Participant demographics 19
F. Dog demographics 21
G. Characteristics of prospectives owners and sources of information utilised 22
H. Themes related to pre-acquisition research 24

A. “Choosing My Dog” survey questions relevant to pre-acquisition research

N.B. Here, questions are grouped by topic and are not necessarily in the order they appeared to survey participants. Not all participants were asked every question: responses to previous questions was used to drive logical progression through aspects of the survey, i.e. those participants who did not own a dog were not asked questions about their dog. An asterisk (*) denotes mandatory questions (i.e. participants were required to give a response before they could progress to the next part of the survey). Responses next to white circles (○) denote where only one answer could be selected. Multiple responses could be selected where they are next to a white square (□). Question text enclosed in square brackets ([]) denotes where an automated, individual question would be included, based on a respondents’ previous response to a question (typically, their dog’s name).

Current and future dog ownership status (ALL respondents)

Question	Response options
Do you currently own a dog? *	○ Yes: I currently own at least one dog<br>○ No: I don't own any dogs at the moment
Are you seriously considering buying or adopting a new dog soon? *	○ Yes: I'm actively looking for a new dog at the moment<br>○ Yes: I am seriously considering getting a new dog in the next six months but I'm not actively looking at the moment<br>○ Yes: I am seriously considering getting a new dog but this probably won't be in the next six months<br>○ No: I'm not currently considering getting a new dog

Dog demographic variables (CURRENT dog owners)

Question	Response options
Which of these best describes [your dog]? *	○ Specific breed (e.g. Labrador Retriever, Whippet)<br>○ Mix of two specific breeds (e.g. Labradoodle, Pug x Beagle)<br>○ Mix of unspecified breeds or unknown
What breed is [your dog]? *	○ [List of common breeds]<br>○ Breed not listed (please specify) [Free text]
What mix of two specific breeds is [your dog]? *	○ [List of common mixes]<br>○ Mix of breeds not listed (please specify) [Free text]
Is [your dog] a particular ‘type’ (e.g. Collie type, Labrador cross)? *	○ [List of common mixed breed types]<br>○ Type not listed (please specify) [Free text]
Did you breed [dog’s name] yourself? *	○ Yes (Those who bred their dog were not included in analyses related to this study.)<br>○ No
When did you get [dog’s name]? *	○ Month [Select a number]<br>○ Year [Select a number]

Question	Response options
Where did you get [dog's name] from? *	o A dog breeder (e.g. someone who owns a female dog who has a litter (For analyses, responses (including free text ones) were grouped into 4 categories: breeder; charity or rehoming centre; family, friends, or community; and private or third party seller.)<br>o Charity/rehoming centre in the UK which rehomes UK dogs only<br>o Charity/rehoming centre in the UK which rehomes dogs from overseas<br>o Charity/rehoming centre based overseas<br>o Friends or family who bred my dog themselves<br>o Friends or family who did not breed my dog themselves<br>o Pet shop<br>o Private/third party seller (e.g. someone selling a puppy or adult dog that they did not breed)<br>o Someone who was giving a dog away for free<br>o Other (please specify) [Free text]

Previous dog ownership experience (CURRENT dog owners)

**Question**	**Response options**
Is this the first time you've lived with a dog?*	o Yes: this is the first time I've lived with a dog<br>o No: I've previously lived with a dog/dogs as an adult<br>o No: I've previously lived with a dog/dogs as a child<br>o No: I've previously lived with a dog/dogs as an adult and as a child
Is this the first time you've lived with this breed or type of dog?*	o Yes: this is the first time I've lived with this breed or type of dog<br>o No: I've previously lived with this breed or type of dog as an adult<br>o No: I've previously lived with this breed or type of dog as a child<br>o No: I've previously lived with this breed or type of dog as an adult and as a child

Considerations prior to acquiring a dog (CURRENT dog owners)

**Question**	**Response options**
Did you consider any of the following when deciding whether to get a dog?*	Select *Considered* or *Not considered* for each item.<br>o The cost of looking after a dog<br>o The time needed to look after a dog<br>o Whether my home has a suitable outdoor space for a dog<br>o Whether I have places to exercise a dog close by

Pre-acquisition research (CURRENT dog owners)

**Question**	**Response options**
Did you look for any information or ask anyone for advice before getting a dog?*	o Yes<br>o No

What information or advice did you look for before getting your dog? *	[Free text]
How did you look for information or advice before getting your dog?*	☐ I discussed getting a dog with family and friends (The first two responses ("*I discussed getting a dog with family and friends*" and "*I asked family or friends for advice*" were combined in analyses.)<br>☐ I asked family and friends for advice<br>☐ I asked a vet for advice<br>☐ I asked a dog behaviorist or training professional for advice<br>☐ I asked a dog walker or dog sitter for advice<br>☐ I asked a dog groomer for advice<br>☐ I asked a dog breeder for advice<br>☐ I asked a member of the public, e.g. while out walking<br>☐ I visited dog events, e.g. Discover Dogs<br>☐ I asked for and/or read advice on online forums<br>☐ I asked for and/or read advice on social media<br>☐ I visited websites to find information<br>☐ I visited blogs to find information<br>☐ I watched TV shows to find information<br>☐ I read books to find information<br>☐ I read magazines to find information<br>☐ None of the above<br>☐ Other (please specify) [Free text]
Please give details of any sources of information you remember using, e.g. websites, books, etc.	[Free text]
Approximately how long did you spend looking for	○ A few hours<br>○ A day<br>○ A week

information before getting your dog?*	○ A few weeks<br>○ A month<br>○ A few months<br>○ Six months<br>○ A year<br>○ Longer than a year<br>○ Other (please specify) [Free text]
Were you able to find all the information you wanted?*	○ Yes: I found all the information I wanted<br>○ No: I didn't find all the information I wanted
What information could you not find? *	[Free text]

Post-acquisition reflections (CURRENT dog owners)

Question	Response options
What advice would you give to potential dog owners about buying or rehoming a dog?	[Free text]

Previous dog ownership experience (POTENTIAL dog owners)

Question	Response options
Have you lived with a dog before?*	○ No: I've never lived with a dog before<br>○ Yes: I've previously lived with a dog/dogs as an adult<br>○ Yes: I've previously lived with a dog/dogs as a child<br>○ Yes: I've previously lived with a dog/dogs as an adult and as a child

Considerations prior to acquiring a dog (POTENTIAL dog owners)

Question	Response options
Have you consider any of the following when deciding whether to get a dog?*	Select *Considered* or *Not considered* for each item.<br>○ The cost of looking after a dog<br>○ The time needed to look after a dog<br>○ Whether my home has a suitable outdoor space for a dog<br>○ Whether I have places to exercise a dog close by

Pre-acquisition research (POTENTIAL dog owners)

Question	Response options
Have you looked for any information or asked anyone for	○ Yes<br>○ No but I plan to<br>○ No and I don't plan to<br>○ I haven't thought about this

advice about getting a dog?*	

Pre-acquisition research (POTENTIAL dog owners who had already undertaken research)

Question	Response options
What information or advice did you want about getting a dog? *	[Free text]
How have you looked for information or asked for advice about getting a dog?*	I discussed getting a dog with family and friends (The first two responses ("*I discussed getting a dog with family and friends*" and "*I asked family or friends for advice*" were combined in analyses.)<br>I asked family and friends for advice<br>I asked a vet for advice<br>I asked a dog behaviorist or training professional for advice<br>I asked a dog walker or dog sitter for advice<br>I asked a dog groomer for advice<br>I asked a dog breeder for advice<br>I asked a member of the public, e.g. while out walking<br>I visited dog events, e.g. Discover Dogs<br>I asked for and/or read advice on online forums<br>I asked for and/or read advice on social media<br>I visited websites to find information<br>I visited blogs to find information<br>I watched TV shows to find information<br>I read books to find information<br>I read magazines to find information<br>None of the above<br>Other (please specify) [Free text]
Please give details of any sources of information you remember using, e.g. websites, books, etc.	[Free text]

Approximately how long have you been looking for information about getting a dog?*	○ A few hours<br>○ A day<br>○ A week<br>○ A few weeks<br>○ A month<br>○ A few months<br>○ Six months<br>○ A year<br>○ Longer than a year<br>○ Other (please specify) [Free text]
Were you able to find all the information you wanted?*	○ Yes: I found all the information I wanted<br>○ No: I didn't find all the information I wanted
What information could you not find? *	[Free text]

Pre-acquisition research (POTENTIAL dog owners who plan to do some research)

Question	Response options
What information or advice do you want about getting a dog? *	[Free text]
How will you look for information or who will you ask for advice before getting a dog?*	☐ I will discuss getting a dog with family and friends (The first two responses ("*I will discuss getting a dog with family and friends*" and "*I will ask family or friends for advice*" were combined in analyses.)<br>☐ I will ask family and friends for advice<br>☐ I will ask a vet for advice<br>☐ I will ask a dog behaviorist or training professional for advice<br>☐ I will ask a dog walker or dog sitter for advice<br>☐ I will ask a dog groomer for advice<br>☐ I will ask a dog breeder for advice<br>☐ I will ask a member of the public, e.g. while out walking<br>☐ I will visit dog events, e.g. Discover Dogs<br>☐ I will ask for and/or read advice on online forums

	- [ ] I will ask for and/or read advice on social media<br>- [ ] I will visit websites to find information<br>- [ ] I will visit blogs to find information<br>- [ ] I will watch TV shows to find information<br>- [ ] I will read a book to find information<br>- [ ] I will read a magazine to find information<br>- [ ] I haven't thought about this<br>- [ ] Other (please specify) [Free text]
Please give details of any sources of information you plan to use, e.g. websites, books, etc.	[Free text]
Approximately how long do you think it will take you to look for information about getting a dog?*	o A few hours<br>o A day<br>o A week<br>o A few weeks<br>o A month<br>o A few months<br>o Six months<br>o A year<br>o Longer than a year<br>o Other (please specify) [Free text]

Participant demographic variables (ALL respondents)

Question	Response options
What is your gender?*	o Female<br>o Male<br>o Prefer not to say<br>o Prefer to self-identify (please specify) [Free text]
What age group do you fit into?*	o 18 - 24 years<br>o 25 - 34 years<br>o 35 - 44 years<br>o 45 - 54 years<br>o 55 - 64 years<br>o 65 - 74 years<br>o 75 - 84 years

	o 85 years or older<br>o Prefer not to say
What is the first part of your postcode? Please leave blank if you would prefer not to say.	[Free text] (Used to group by nation.)
What is your highest level of qualification/education?*	o No formal qualifications<br>o GCSE/National 5 or equivalent<br>o A level/Scottish Higher or equivalent<br>o Foundation degree/Higher National Diploma (HND) or equivalent<br>o University degree (e.g. BA, BSc) or equivalent<br>o Post graduate degree (e.g. MA, MBA, MSc, PhD) or equivalent<br>o Prefer not to say
Have you ever worked with dogs?*	o Yes: I currently work with dogs<br>o Yes: I've previously worked with dogs but I don't at the moment<br>o No: I've never worked with dogs<br>o N/A: I've never worked<br>o Prefer not to say<br>☐ Please give more details if you would like to [Free text]
How did you find out about this survey?	o Colleague<br>o Dogs Trust charity shop<br>o Dogs Trust Contact Centre (phone/email)<br>o Dogs Trust Dog School<br>o Dogs Trust e-newsletter<br>o Dogs Trust event<br>o Dogs Trust rehoming centre<br>o Dogs Trust WAG magazine<br>o Friend or family<br>o Local press<br>o Search engine<br>o Social media (e.g. Facebook, Twitter)<br>o Prefer to self-identify (please specify) [Free text]
Do you have children under 18 living at home with you, or who you have regular responsibility for?*	o Yes<br>o No<br>o Prefer not to say
What is your employment status?*	o Employed full time (35 hours or more per week)<br>o Employed part time (less than 35 hours per week)<br>o Self employed full time (35 hours or more per week)

- Self employed part time (less than 35 hours per week)
- Unemployed and currently looking for work
- Unemployed and not currently looking for work
- Student
- Retired
- Homemaker/housewife/househusband
- Unable to work
- Prefer not to say

What is your annual household income?*

- Less than £15,000
- £15,000 to £24,999
- £25,000 to £34,999
- £35,000 to £44,999
- £45,000 to £54,999
- £55,000 to £64,999
- £65,000 to £74,999
- £75,000 to £84,999
- £85,000 to £94,999
- £95,000 to £104,999
- £105,000 or more
- N/A: no one in my household works
- Prefer not to say

B. "Choosing My Dog" interview schedule

N.B. These are the complete set of questions for *current* and *potential* dog owners (i.e. those who already owned a dog and were thinking about getting another dog). Interviews were semi-structured so not all owners were asked all questions. For interviews with *current* (not *potential*) owners, most later questions were omitted or amended whether appropriate. For interviews with *potential* (not *current*) owners, most earlier questions were omitted or amended where appropriate. Questions next to white circles (○) were prompt questions hence not all these questions were asked to every participant. Text enclosed in square brackets ([]) refers to guidance and notes for the interviewer.

Interviews were conducted between April 2019 and March 2020. Participants were current (n=24) and potential (n=8) dog owners. Interviewees were recruited through the survey (n=15), pilot survey (n=5), or were members of Dogs Trust staff (n=12). Interviews were conducted remotely via telephone (n=22) or face-to-face (n=10). The majority of interviews were with individuals (n=25) but seven individuals were involved in group interviews with two or three participants in each Interviews lasted between 17 and 60 minutes in length (mean=33 minutes, median=29 minutes).

Opening (introductions)

[Thank participants, provide a brief overview of how the interview will run. Explanation of why we will be recording the interview and confirmation of consent. Check whether there are any questions then start recording.]

Section A – About your dog

- Can you tell me about your dog?
    - ○ What is their name?
    - ○ How old are they?
    - ○ What breed or type of dog are they?
- How long have you had your dog?
    - ○ How old were they when you got them?
- What is your dog's daily routine?
    - ○ Who in your household looks after your dog?

Section B – Deciding to get a dog

- Is this the first time you've owned a dog?
    - [If multiple previous dogs it might be useful to make a brief timeline/note all dogs]
- Can you tell me about your previous experiences with dogs?
    - ○ What breeds(s) or types?
    - ○ Were these experiences when you were a child or an adult?
    - ○ How did you get your previous dog(s)
- Who made the decision to get [this dog]?
- How long had you been thinking about getting a dog?
    - ○ How long had you been thinking about getting [this dog]?

    - What prompted you to act when you did?
- Can you describe why you wanted to have a dog?
    - What were the important reasons behind you wanting to have a dog (in general)?
    - How did you imagine having a dog would affect your life (and that of your household)?
    - Did you have any concerns about getting a dog?

Section C – Before finding your dog

- You'd decided you wanted a dog: what did you do next?
    - Did you already have a clear idea of what you wanted?
        - Did you know what breed or type of dog you'd like?
        - Did you consider a range of breeds?
        - What about age of dog?
        - Did you have any ideas about where you wanted to get your dog from?
- Did you look for any information or ask anyone for advice before getting your dog?
    - Who did you ask?
    - Where did you look?
    - What information did you want to find?
    - Were you able to find that information?
    - Was there any information you couldn't find but you would have liked?
    - Approximately how long did you spend looking for information?
        - Was it a long or short process?
- What were important factors when trying to narrow down which breed or type of dog you wanted [if not already covered]?
    - What attracted you to a particular breed or type?
        - Had you had any previous experiences with the breed/type?
        - E.g. family/friends owned this breed/type?
    - What do you like about this particular breed?
- Did you change your mind or have any second thoughts during this process?
    - Was it an easy decision or did you rethink anything?
    - What did you do as a result of any concerns?

Section D – Finding your dog

- Where did you get [your dog] from?
    - Was it a breeder/rehoming centre, etc.
        - Did this matter to you at the time?
            - Why/why not?
    - Why did you choose this source?
    - How did you find this source?
    - Was this the only place you looked?
    - [If no, probe more about other dogs they may have enquired about, e.g.]
        - Did you see any other dogs you liked?
        - Did you speaker to the seller/rescue?

        - What happened?
        - Why did you decide against that dog?
    - What other sources did you consider?
        - What process did you go through to look for possible sources?
        - How did you know where to look?
- When was the first time you met [your dog]?
    - What was the place like?
    - What happened?
        - If [your dog] was a puppy, did you meet your dog's mother/siblings?
    - What questions did you have (if any)?
    - Were there any negatives about that experience?
        - Was there anything that made you have second thoughts or want to ask more questions?
    - How did you feel?
- How did you decide on [your dog] [if not already covered]?
    - Did you already have a clear idea of what you wanted?
- How many times did you meet your dog before you took them home?
    - Over what period of time?
- What did your dog come home with?
    - Was there any after care offered?
    - E.g. Would any support be offered if there were any issues?
- Who was involved in the process?
    - E.g. household member/family?
        - [If not already know, ask about their previous experiences with dogs]
    - How were they involved in the process?
    - Was it a long or short process?
    - Was it easy or difficult?
    - How did you feel?
- Was it an easy decision or did you rethink anything?
    - Did you change your mind during this process?
    - What did you do as a result of any concerns
- How long overall do you think it took you to get [your dog]?

Section E – After acquiring your dog

- How does [your dog] compare to the dog you thought you'd have, before you got them?
    - E.g. Is the breed or type of dog what you imagined yourself with?
- What did you imagine life with [your dog] would be like, before you got them?
    - Has it lived up to those expectations?
    - What changes has having a dog made to your life?
    - What have you most enjoyed?
    - Is there anything that's been different to what you expected?
- Would you recommend this breed or type of dog to potential owners?
    - Why/why not?

- Would you recommend the source you acquired your dog from to potential owners?
    - Why/why not?
- If you were looking for another dog, where would you look now?
- In hindsight, do you feel that you spent enough time making the decision to get a dog?
    - Did you have all the information you needed?
- What advice would you give to other potential dog owners?

Section F – Deciding to get a(nother) dog

- You're planning to get a(nother) dog! Who made the decision to get a(nother) dog?
- How long have you been thinking about getting a(nother) dog?
    - What prompted you to act when you did?
- Can you describe why you want to have a(nother) dog?
    - How do you imagine having another dog will affect your life (and that of your household, including your current dogs)?
    - Do you have any concerns about getting a(nother) dog?

Section G – Before getting another dog

- You decided that you want a(nother) dog: what did you do first?
    - Did you already have a clear idea of what you wanted?
        - Did you know what breed or type of dog you'd like?
        - Did you consider a range of breeds?
        - What about age of dog?
        - Did you have any ideas about where you want to get your dog from?
            - Why?
- What were important factors when trying to narrow down which breed or type of dog you wanted [if not already covered]?
    - What attracted you to a particular breed or type?
    - What do you like about this particular breed?
- Have you looked for any information or asked anyone for advice?
    - Who did you ask?
    - Where did you look?
    - What information did you want to find?
    - Were you able to find that information?
    - Was there any information you couldn't find but you would have liked?
    - Approximately how long did you spend looking for information?
        - Was it a long or short process?
- Have you changed you mind about what you initially wanted?
    - Why do think this is?
- Who else has been involved in this process?
    - E.g. household member/family?
    - Has it been easy or difficult?

Section H – Looking for a(nother) dog

- What are you doing at the moment?
    - Are you actively looking for a(nother) dog?
    - What have you done in the last… day? / week?
    - What do you plan to do in the next week?
- Have you seen any dogs that you thought might be right for you?
    - What did you like about them?
    - Where did you see them? E.g. online?
    - Were they from a breeder/rehoming centre, etc.
        - Did this matter to you at the time?
            - Why/why not?
    - What did you do?
    - What happened?
    - What changed you mind? / Why didn't
- Have you met any dogs?
    - When was this?
    - What happened?
    - How did you find this source?
    - Was this the only place you looked?
- Have you considered any other sources?
    - What process did you go through to look for possible sources?
    - How did you know where to look?
    - What was your experience with (this sources/these sources) like?
- Have you changed your mind or had any second thoughts during this process?
    - Was it an easy decision or have you rethought anything?
    - What did you do as a result of any concerns?
- Given your experiences, what advice would you give to other potential dog owners?

Ending (brief summary of main points)

- Is there anything else we should have talked about but didn't – related to getting a dog?
- Have we missed anything that you think is important?
- Thank you so much for your time and sharing your experiences with us. This has been really interesting and is very important for our work. Thank you!

C. Participant recruitment

Supplementary Table S1. Participant recruitment (current owners n=4381, potential owners n=2350).

How participant found out about the survey	Current (n=4381)		Potential (n=2350)	
	n	%	n	%
Social media	2583	58.96%	927	39.45%
Dogs Trust Contact Centre	362	8.26%	890	37.87%
Dogs Trust e-newsletter	570	13.01%	111	4.72%
Dogs Trust WAG magazine	306	6.98%	90	3.83%
Friend or family	115	1.43%	127	4.40%
Other	496	11.32%	230	9.79%

D. Coding of survey free text responses

Supplementary Table S2. Coding of survey free text responses. Note “Number of responses” represents the number of respondents this question was shown to and does not necessarily mean that this number of respondents gave a valid response.

Open-ended survey question	Ownership Status	Pre-acquisition research status	Number of respondents asked	Coding completed
What information or advice did you look for before getting your dog?	Current	Completed research	4381	● Responses 1-1000: all coded (R.M.)<br>● Responses 1001-2000: all coded (K.E.H.) plus every 25th response coded independent (R.M.)<br>● Responses 2001-4381: every 25th response coded (R.M.)
How did you look for information or ask for advice before getting your dog? ("Other")	Current	Completed research	375	● Responses 1-375: all coded (R.M.)
Please give details of any sources of information you remember using.	Current	Completed research	4381	● Responses 1-1000: all coded (R.M.)<br>● Responses 1001-2000: all coded (K.E.H.) plus every 25th response coded independently (R.M.)<br>● Responses 2001-4381: every 25th response coded (R.M.)
Approximately how long did you spend looking for information before getting your dog? (“Other”)	Current	Completed research	65	● Responses 1-65: all coded (R.M.)
What information could you not find?	Current	Completed research	187	● Responses 1-187: all coded (R.M.)
What information or advice did you look for before getting your dog?	Potential	Completed research	1955	● Responses 1-1000: all coded (R.M.)<br>● Responses 1001-1955: all coded (K.H.) plus every 25th response coded independently (R.M.)
How did you look for information or ask for advice before getting your dog? ("Other")	Potential	Completed research	199	● Responses 1-199: all coded (R.M.)
Please give details of any sources of information you remember using	Potential	Completed research	1955	● Responses 1-1000: all coded (R.M.)<br>● Responses 1001-1955: all coded (K.E.H.) plus every 25th response coded independently (R.M.)
Approximately how long have you been looking for information about getting a dog? (“Other”)	Potential	Completed research	64	● Responses 1-64: all coded (R.M.)

What information could you not find?	Potential	Completed research	192	• Responses 1-192: all coded (R.M.)
What information or advice do you want about getting a dog?	Potential	Plan to undertake research	395	• Responses 1-386: all coded (R.M.)
How will you look for information or who will you ask for advice before getting a dog? ("Other")	Potential	Plan to undertake research	57	• Responses 1-57: all coded (R.M.)
Approximately how long do you think it will take you to look for information about getting a dog? ("Other")	Potential	Plan to undertake research	18	• Responses 1-18: all coded (R.M.)
Please give details of any sources of information you plan to use.	Potential	Plan to undertake research	395	• Responses 1-386: all coded (R.M.)

E. Participant demographics

Supplementary Table S3. Participant demographics (current owners n=4381, potential owners n=2350).

	Current owners (n=4381)		Potential owners (n=2350)	
	n	%	n	%
**Respondent gender**				
Female	3895	88.91%	1883	80.13%
Male	439	10.02%	444	18.89%
Non-binary	7	0.16%	1	0.04%
Prefer to self-identify	33	0.75%	2	0.09%
Prefer not to say	7	0.16%	20	0.85%
**Respondent age group**				
18 - 24 years	311	7.10%	198	8.43%
25 - 34 years	839	19.15%	507	21.57%
35 - 44 years	731	16.69%	388	16.51%
45 - 54 years	1036	23.65%	457	19.45%
55 - 64 years	904	20.63%	449	19.11%
65 - 74 years	463	10.57%	274	11.66%
75 - 84 years	61	1.39%	55	2.34%
85 years or older	4	0.09%	5	0.21%
Prefer not to say	32	0.73%	17	0.72%
**Region (based on post code)**				
England	3005	68.59%	1622	69.02%
East Midlands	215	4.91%	101	4.30%
East of England	357	8.15%	157	6.68%
Greater London	218	4.98%	146	6.21%
North East	187	4.27%	78	3.32%
North West	431	9.84%	242	10.30%
South East	58	1.32%	310	13.19%
South West	371	8.47%	201	8.55%
West Midlands	471	10.75%	204	8.68%
Yorkshire and the Humber	457	10.43%	181	7.70%
Northern Ireland	58	1.32%	31	1.32%
Scotland	371	8.47%	203	8.64%
Wales	171	3.90%	103	4.38%
No response	776	17.71%	391	16.64%
**Highest level of qualification/education**				
No formal qualifications	107	2.44%	90	3.83%
GCSE/National 5 or equivalent	656	14.97%	390	16.60%
A level/Scottish Higher or equivalent	525	11.98%	271	11.53%
Foundation degree/Higher National Diploma (HND) or equivalent	570	13.01%	299	12.72%
University degree (e.g. BA, BSc) or equivalent	1362	31.09%	668	28.43%

Post graduate degree (e.g. MA, MBA, MSc, PhD) or equivalent	831	18.97%	404	17.19%
Prefer not to say/no response	330	7.53%	228	9.70%
**History with dogs**				
Previously lived with a dog/dogs as an adult and as a child	1588	36.25%	1087	46.26%
Previously lived with a dog/dogs as an adult	1236	28.21%	690	29.36%
Previously lived with a dog/dogs as a child	850	19.40%	286	12.17%
First time lived with a dog	707	16.14%	287	12.21%
**Worked with dogs**				
Currently work with dogs	386	8.81%	133	5.66%
Previously worked with dogs	380	8.67%	275	11.70%
Never worked with dogs	3409	77.81%	1813	77.15%
N/A: never worked	98	2.24%	64	2.72%
Prefer not to say/no response	108	27.98%	65	48.87%
**Children (<18) living at home**				
No	3470	79.21%	1844	78.47%
Yes	863	19.70%	492	20.94%
Prefer not to say	48	1.10%	14	0.60%
**Employment status**				
Employed full time (35 hours or more per week)	1760	40.17%	932	39.66%
Employed part time (less than 35 hours per week)	748	17.07%	417	17.74%
Self employed full time (35 hours or more per week)	221	5.04%	73	3.11%
Self employed part time (less than 35 hours per week)	227	5.18%	106	4.51%
Unemployed and currently looking for work	25	0.57%	33	1.40%
Unemployed and not currently looking for work	26	0.59%	21	0.89%
Student	106	2.42%	76	3.23%
Retired	840	19.17%	454	19.32%
Homemaker/housewife/househusband	168	3.83%	76	3.23%
Unable to work	89	2.03%	65	2.77%
Prefer not to say/no response	171	3.90%	97	4.13%
**Annual household income**				
Less than £15,000	257	5.87%	167	7.11%
£15,000 to £24,999	493	11.25%	347	14.77%
£25,000 to £34,999	500	11.41%	342	14.55%
£35,000 to £44,999	471	10.75%	251	10.68%
£45,000 to £54,999	381	8.70%	200	8.51%
£55,000 to £64,999	304	6.94%	135	5.74%
£65,000 to £74,999	245	5.59%	113	4.81%
£75,000 to £84,999	188	4.29%	86	3.66%
£85,000 to £94,999	109	2.49%	35	1.49%
£95,000 to £104,999	100	2.28%	32	1.36%
£105,000 or more	198	4.52%	88	3.74%
N/A: no one in household works	35	0.80%	22	0.94%
Prefer not to say/no response	1100	25.11%	532	22.64%

F. Dog demographics

Supplementary Table S4. Dog demographics (current owners, n=4381).

	n	%
**Source of dog**		
Charity/rehoming centre	2125	48.50%
A dog breeder	1655	37.78%
Friends or family/community	393	8.97%
Private/third party seller	208	4.75%
**Breed or type of dog**		
Specific breed	2525	57.64%
Mix of two specific breeds	1012	23.10%
Mix of breeds or a type	844	19.27%
**Age of dog at acquisition**		
Puppy (<=6 months)	2673	61.01%
Juvenile (7-<12 months)	235	5.36%
Young adult (1-<2 years)	391	8.92%
Mature adult (2-6 years)	857	19.56%
Senior adult (7-11 years)	195	4.45%
Geriatric (>=12 years)	30	0.68%
**Year of acquisition**		
2001-2010	528	12.05%
2011	173	3.95%
2012	195	4.45%
2013	279	6.37%
2014	309	7.05%
2015	351	8.01%
2016	452	10.32%
2017	563	12.85%
2018	739	16.87%
2019	790	18.03%
No response	2	0.05%

G. Characteristics of prospectives owners and sources of information utilised

Supplementary Table S5 Source of information for current owners who undertook research by age category, n=4381 (respondents could select multiple responses.).

Source	18 - 24 years	25 - 34 years	35 - 44 years	45 - 54 years	55 - 64 years	65 - 74 years	75 years or older	Prefer not to say
Family or friends	79.74%	81.29%	71.00%	67.28%	64.71%	56.37%	56.92%	68.75%
Websites	77.81%	83.19%	80.16%	76.25%	72.35%	66.52%	60.00%	71.88%
Books	32.15%	36.00%	38.85%	37.93%	39.49%	36.50%	24.62%	37.50%
Online forums	63.99%	61.03%	57.59%	49.42%	45.24%	34.56%	20.00%	43.75%
Social media	41.16%	33.61%	31.05%	25.58%	18.81%	13.39%	3.08%	21.88%
TV	18.65%	15.61%	15.05%	12.45%	10.51%	7.34%	0.00%	15.63%
Vet	16.72%	12.87%	14.50%	13.51%	13.94%	12.96%	6.15%	21.88%
Blogs	27.65%	24.08%	19.84%	11.00%	6.31%	4.32%	1.54%	9.38%
Dog breeder	15.43%	15.73%	16.96%	15.73%	17.48%	18.14%	20.00%	15.63%
Dog behaviourist/training professional	16.40%	14.42%	14.64%	10.42%	10.07%	10.37%	10.77%	15.63%
Events	9.32%	10.85%	11.49%	9.85%	11.06%	7.13%	7.69%	9.38%
Member of public	9.65%	7.75%	9.44%	9.17%	5.75%	7.78%	6.15%	12.50%
Magazines	9.97%	6.44%	9.44%	8.88%	10.18%	10.58%	9.23%	12.50%
Dog walker/sitter	4.50%	6.44%	6.84%	6.56%	3.32%	5.18%	7.69%	6.25%
Dog groomer	3.54%	2.62%	1.64%	2.90%	2.21%	2.59%	3.08%	0.00%
None of the above	1.67%	2.46%	4.05%	4.42%	7.34%	9.84%	9.23%	3.65%

Supplementary Table S6. Source of information for current owners who undertook research by gender, n=4381 (respondents could select multiple responses).

Source	Female	Male	Self-identify	Prefer not to say
Family or friends	70.12%	66.51%	57.14%	60.61%
Websites	76.30%	75.40%	71.43%	81.82%
Books	37.66%	33.71%	35.71%	39.39%
Online forums	50.94%	51.94%	50.00%	63.64%
Social media	25.85%	27.79%	35.71%	27.27%
TV	12.66%	14.58%	0.00%	15.15%
Vet	13.92%	12.30%	7.14%	18.18%
Blogs	14.20%	15.49%	7.14%	18.18%
Dog breeder	16.64%	16.40%	21.43%	12.12%
Dog behaviourist/training professional	12.32%	11.85%	7.14%	15.15%
Events	10.24%	8.66%	28.57%	18.18%
Member of public	8.34%	6.15%	7.14%	6.06%
Magazines	9.14%	8.88%	0.00%	6.06%
Dog walker/sitter	5.75%	4.78%	7.14%	3.03%
Dog groomer	2.49%	2.51%	7.14%	0.00%
None of the above	3.77%	2.96%	0.00%	0.00%

Supplementary Table S7. Source of information for current owners who undertook research by source dog was acquired from, n=4381 (respondents could select multiple responses).

Source	A dog breeder	Charity/rehoming centre	Friends or family/community	Private/third party seller
Family or friends	70.35%	67.13%	75.83%	70.67%
Websites	82.26%	70.45%	64.63%	82.69%
Books	48.42%	23.32%	38.68%	31.73%
Online forums	59.48%	40.91%	46.82%	55.29%
Social media	29.41%	22.42%	22.14%	28.85%
TV	13.22%	12.21%	12.21%	14.90%
Vet	15.53%	10.57%	19.59%	10.10%
Blogs	17.18%	10.94%	12.21%	16.35%
Dog breeder	29.32%	1.75%	15.01%	7.69%
Dog behaviourist/training professional	11.06%	14.38%	12.72%	7.21%
Events	12.42%	8.22%	6.87%	9.62%
Member of public	10.78%	4.71%	6.36%	11.06%
Magazines	10.73%	7.55%	8.14%	5.77%
Dog walker/sitter	5.18%	5.38%	8.40%	7.21%
Dog groomer	3.06%	1.39%	3.31%	3.85%
None of the above	1.18%	7.25%	3.05%	1.44%

H. Themes related to pre-acquisition research

Supplementary Table 8. Themes related to the information prospective dog owners seek prior to acquiring a dog.

**Overarching themes**	**Main themes**	**Sub-Themes**	**Example quotations**
Information about dogs	The needs or requirements of a dog	Activeness or energy	"*Energy levels"* (Current owner, survey ID 2153)
		Exercise	"*Need for exercise and stimulation*." (Potential owner, survey ID 1412)<br>*"Amount of exercise the dog needs."* (Current owner, survey ID 306)
		Feeding or diet	*"Best food to feed them."* (Current owner, survey ID 208)
		Grooming	*"Grooming requirements."* (Current owner, survey ID 145)
		Health	*"Read up on potential diseases."* (Current owner, survey ID 884)<br>*"Health issues & life expectancy."* (Current owner, survey ID 1252)<br>*"Common health conditions."* (Current owner, survey ID 1845)
		Needs<br><br>- Rescue-specific	"*What a dog needs in order to be happy and content*." (Current owner, ID 8022)<br>"*How to provide what the dog needs*." (Potential owner, survey ID 2836)<br>*"I looked at specific info on the needs of rescued puppy farm breeder dogs."* (Current owner, survey ID 1060)
		Space	"*Space needed*." (Current owner, survey ID 686)
	Dog characteristics	Behaviour	"*What behaviour traits to look for*." (Current owner, survey ID 79)
		Personality	"*Personality, attitude, temperament*." (Current owner, survey ID 1958)
		Size	*"Considered size."* (Current owner, survey ID 36)
		Temperament	"*Temperament*." (Current owner, survey ID)
		Trainability	"*Trainability.*" (Potential owner ID 1016)
Information about breeds or types of dogs	Choosing a breed or type of dog	How to choose which breed or type of dog	*"I researched breeds heavily before deciding which would be suitable for me*." (Current owner, survey ID 228)<br>*"Went to Discover Dogs show to talk to various breed societies to work out which breed would be best for us*." (Current owner, survey ID 820)<br>"*I used the Internet a lot to help me choose what breed was best suitable for me. Breed information from the kennel club, and forums with other dog owners mainly. I also discussed it a lot with my mum, as I lived*

			with her and size/breed of dog were an important factor to discuss and agree on." (Current owner, survey ID 2086)<br>*"Looked at questionnaires which matched dog breed to lifestyle."* (Current owner, survey ID 7461)
		Narrowing down potential breeds or types	*"Read up on the breeds we were considering."* (Current owner, survey ID 87)<br>*"We looked at breeds that we liked and weighed up pro's and con's for all, and narrowed it down to two breeds, and then did extensive research on both*." (Current owner, survey ID 1910)
		Specific breed<br><br><br><br><br><br><br><br><br><br><br><br>- Interventions that changed behaviour	*"We did do quite a lot of research about Staffies and what not because none of us have actually ever had Staffs. So, we just wanted to know a little bit about what habits they have. I looked into a lot of things like the best way to train them, certain behaviours, what sort of diets the [sic.] best for them."* (Current owner, interview ID B1RM1104)<br>"*Once we knew we were getting [dog's name]. We did lots of research into dog care in general and further research into his breed. We wanted to ensure we had a good knowledge and understanding of everything he would need in relation to health and well-being specifically. Making sure we knew how to train a dog properly and we knew how to read their body language and assess situations to ensure safety. We were very thorough in finding our everything we could about being a dog owner*." (Current owner, survey ID 1696)<br>"*Once we decided to get Newfoundland's [sic.], we looked up every single possible reason online for not getting a Newfoundland. And considered whether we could live with them; we decided we could*." (Current owner, survey ID 3843)<br>*"We had a home visit from a staffy, which made us realise that is [sic.] wasn't the breed for us. Because we learnt that Staffies love attention and kept active due to the breeds intelligence. And because of our life style we understand that this breed would be unhappy living with us*." (Current owner, survey ID 1208)
	Information about a breed or type	Characteristics, temperament or traits	"*Researched breed characteristics*." (Current owner, survey ID 205)<br>"*Breed trait advice from people with the breed I wanted. What temperament and traits to expect*." (Current owner, survey ID 826)<br>"*Temperament of various breeds*." (Potential owner, survey ID 1040)
		Health<br>- Health issues or illness<br><br><br>- Health tests	"*Health of breed.*" (Current owner, survey ID 2384)<br>"*Known health issues with certain breeds.*" (Potential owner, survey ID 422)<br>"*Any health issues various dogs may encounter (averting my choice from being a pug)*." (Potential owner, survey ID 1871)<br>"*Specific breed related illness and diseases and recommended parent health tests*." (Current owner, survey ID 1195)

			"*What health tests should they have*." (Current owner, survey ID 1692)
		Coat type<br>- Grooming or coat care<br>- Hypoallergic<br><br>- Shedding	"*Coat of breed*." (Current owner, survey ID 685)<br>"*Specific dog breeds, specific grooming needs*." (Potential owner, survey ID 3463)<br>"*Researched on the internet about cavapoo to see if they are hypoallergenic*." (Current owner, survey ID 2)<br>"*I looked online at various breed specifics such as […] shedding hair*." (Current owner, survey ID 1271)
		Training or trainability	"*I looked at info about the breed(exercise, trainability, […] etc*)." (Current owner, survey ID 2012)<br>"*Checked the exercise and training needs for the specific breed we are interested*." (Potential owner, survey ID 1325)
		The needs or requirements of a breed or type	"*I wished to be fully ware [sic.] of the specific needs of this breed to ensure that I was able to meet them*." (Current owner, survey ID 976)
Owner requirements and dog suitability	Family members	Babies<br><br>- Future plans	"*As we were welcoming a baby, we both agreed that we needed to raise a dog we knew we would trust.*" (Current owner, survey ID 1794)<br>"*Are they good with children if I was to have a baby in the future*." (Potential owner, survey ID 1689)
		Children	"*How suitable the dog would be for a family with young children*." (Current owner, survey ID 823)<br>"*I looked at breeds which were good around young children. My previous dog was a Jack Russell and whilst I wanted another one we decided on a spaniel due to their calmer nature*." (Current owner, survey ID 1080)
		Family<br><br><br><br><br>- Future plans	"*Breeds of dogs that were good family pets and had good personalities*." (Current owner, survey ID 904)<br>*"It was also important the dog had a good temperament and would be good with children."* (Current owner, survey ID 1986)<br>"*Internet research on the breed to make sure it would be a good family pet*." (Current owner, survey ID 2210)<br>"*I wanted a family friendly dog as we wanted children*." (Current owner, survey ID 644)
		Allergies / hypoallergic	"*As we had very mild allergies in the house, we looked for a hypo allergenic dog*." (Current owner, survey ID 1132)<br>"*My husband is allergic to many dogs so I researched low allergenic breeds*." Current owner, survey ID 1986)
	House and garden	House size	"*Best size of dog for my home/lifestyle*." (Current owner, survey ID 283)<br>"*If our house is big enough for a dog*." (Potential owner, survey ID 2844)
		Flats or apartments	"*Best small breeds for living in an apartment*." (Current owner, survey ID 436)

			"*Having a dog in a flat with limited outside space*." (Potential owner, survey ID 1523)<br>"*Would I be able to adopt a dog living in a flat? I am allowed a dog & there is use of a communal garden*." (Potential owner, survey ID 9410)
		Potential adjustments for a dog	*"The suitability of our house and garden for a dog, whether we needed to change anything there."* (Potential owner, survey ID 1325)
	Lifestyle	Suit lifestyle	"*Researched how lifestyle would suit different breeds*." (Current owner, survey ID 205)<br>"*I looked to see if this type of dog would suit my lifestyle and be a good fit for me and me for them*." (Current owner, survey ID 1632)
		Active	*"I wanted an active dog who would be able to enjoy long walks and activities such as agility*." (Current owner, survey ID 1805)
		Work	"*Would I be able to look after the puppy while working? Would he be able to come to work with me?*" (Current owner, survey ID 956)
		- Questioning working and having a dog<br>- Time alone<br>- Consideration of support when home alone	*"How working and having a dog can work."* (Potential owner, survey ID 624)<br>"*Whether it's appropriate when you work full time*." (Potential owner, survey ID 1627)<br>*"Is it fair to the dog with the hours we work."* (Potential owner, survey ID 3194)<br>"*Time they can be left alone while I'm at work*." (Potential owner, survey ID 2605)<br>"*If it was possible to work full time and keep a dog properly, if walking services/ daycare were sufficient to prevent anxiety or boredom.*" (Potential owner, survey ID 2465)
	Other animals	Multi dog households<br>- Introducing a second dog<br>- Match for other dog(s)	"*Advice on having a second dog in our home*." (Current owner, survey ID 1755)<br>"*Multi dog households, how to keep a small dog safe around larger dogs, how to prevent little dog syndrome*." (Current owner, survey ID 2084)<br>"*Introducing a new dog to current dog*." (Current owner, survey ID 630)<br>"*Researched on introducing a new dog to a home with established dogs*." (Current owner, survey ID 1225)<br>"B*reed, age and sex to mix with our existing dog*." (Current owner, survey ID 321)<br>"*Choosing a suitable match for resident dog*." (Current owner, survey ID 1078)
		Other pets<br>- Cats	"*Compatability [sic.] with my other pets*." (Current owner, survey ID 2223)<br>"*I have four pet chickens so I wanted to find out which dogs have least prey drive*." (Potential owner, survey ID 3591)<br>"*Advice on […] introducing a dog to my two cats*." (Current owner, survey ID 278)<br>"*Which breeds are best with cats*." (Current owner, survey ID 711)

			"*Googling 'what is the best breed of dog to live with cats' and reading the subsequent selections*." (Current owner, survey ID 3412)
	Match for each other	Owner and dog are a good match for each other	"*I looked into the breed to ensure we would be right for each other.*" (Current owner, survey ID 386)<br>"*How to find a perfect match for the dog with the home and circumstances I have to offer. The match is my overall priority and concern.*" (Potential owner, survey ID 1223)
Aspects of dog ownership	Having a dog	What having a dog is like	"*Just how it would be having a dog*." (Current owner, survey ID 705)<br>*"We spoke to someone who fostered for a rescue and had always had dogs. She spoke to us about the experience of having a dog and the time and commitment needed."* (Current owner, survey ID 1399)<br>"*Reality of dog ownership.*" (Current owner, survey ID 10978)
		How life may change<br>- Lifestyle<br>- Including limitations	"*How life changes*." (Current owner, survey ID 1912)<br>*"How friends and family have found their lifestyle altered by having a dog, both in positive and negative ways."* (Potential owner, survey ID 1158)<br>"*Changes required in lifestyle, limitations that dog ownership might bring e.g. travel, holiday accommodation etc*." (Potential owner, survey ID 3556)
		Commitment and responsibility<br>- Time	"*The commitment of a dog/costs*." (Current owner, survey ID 8022)<br>"*We ended up fostering initially for a animal charity to ensure we could handle the responsibility before we made the ultimate commitment*." (Current owner, survey ID 11226)<br>"*Spoke to my parents who own a dog about responsibilities, cost and time that we'd need to put into having a dog*." (Current owner, survey ID 1806)<br>"*I have researched the breed, and thought about the time I could give my dog*." (Potential owner, survey ID 553)
	Caring for a dog	Caring for a dog	"*General day to day looking after a dog*." (Current owner, survey ID 3161)
		Equipment	*"Research onling [sic.] about what items to get the dog and how to get the home ready*." (Current owner, survey ID 1837)
		Insurance	*"Best insurance companies.*" (Potential owner, survey ID 660)<br>"*Insurance type/cost*." (Current owner, survey ID 1487)
		Vaccinations and vet care	"*Which insurance and vets is best*." (Current owner, survey ID 208)<br>"*Vaccinations and vet care costs.*" (Current owner, survey ID 870)
		Rescue-specific	"*Needs particular to rescues*." (Potential owner, survey ID 470)<br>"*How to care to the specific needs of rescued ex-street dogs who haven't experienced life in a home before*." (Current owner, survey ID 1614)

	Costs	Costs of having a dog	"*We did lots of research on the costs of having a dog, e.g. food, dog walkers, etc*." (Current owner, survey ID 392)
			"*Approximate insurance, vet services and food costs."* (Potential owner, survey ID 2316)
		Dog care/walkers	"*Cost of walkers and daycare*." (Current owner, survey ID 486)
			"*The cost and availability of dog walkers in the local area*." (Potential owner, survey ID 1325)
		Food	"*Types of food to use and cost*." (Current owner, survey ID 616)
		Insurance	"*Cost of insurance*." (Current owner, survey ID 34)
		Vets	*"Considered all aspects of dog ownership before even beginning to look; financial, including which of 3 local vet practises had the best plans and coverage*." (Current owner, survey ID 730)
	Support network	Dog care<br><br>- Day care<br><br><br>- Dog walkers and sitters	"*I have also spoken to local dog cares to ensure I have a support network in place if I needed someone else to look after my dog*." (Potential owner, survey ID 4496)<br><br>"*Will the day care arrangements be enough to keep a young dog happy and settled, is the associated expense of having 2 dogs in full time daycare affordable?"* (Current owner, survey ID 956)<br><br>"*Viewed several daycare providers*." (Current owner, survey ID 450)<br><br>"*I googled and searched Facebook for dog walkers and home boarders, then followed up their references and asked around*." (Current owner, survey ID 3258)<br><br>"*I want to know how people balance their work hours with owning a dog and how much they have to spend on dog walkers etc. I also worry about finding a trustworthy dog walker. I have worked in that industry and know so many other people I wouldn't be happy leaving my dog with - even if they have the best intentions they really don't know how to look after dogs and take too many risks (don't know first aid, don't know dog body language, walk too many dogs at once, multiple dogs on flexileads)."* (Potential owner, survey ID 1)
		Family	"*What support would I have from family for when I was away?*" (Current owner, survey ID 956)
		Friends	"*Friends willing to care for the dog, when on holiday*." (Current owner, survey ID 1814)
		Parents	"*Had to check my parents could look after her while I'm at work*." (Current owner, survey ID 329)
		Unexpected<br><br>- Longer term	"*Backup people and companies for if I unexpectedly can't look after a pet."* (Potential owner, survey ID 470)<br>*"Provisions in relation to care of my dogs in the case of my death, or long periods of hospitalisation."* (Potential owner, survey ID 6682)

	Training	Training	"*Information about puppy socialisation, training classes and positive reinforcement training techniques and programmes, further enrichment training for adult dogs like agility and scent classes*." (Potential owner, survey ID 1798)<br>"*Training videos online. Spoke to friends with dogs regarding training, potty training, sleep etc*." (Current owner, survey ID 2001)
		Puppy	"*Read books on training a puppy. Advice about local puppy training classes*." (Current owner, survey ID 1127)
		Training classes<br>- Visited prior to acquiring a dog	"*Researched local training classes.*" (Current owner, survey ID 9751)<br>"*Before getting our 1st dog , went to a local training club to watch the classes and see what was involved in training a dog to become a well behaved member of society.*" (Current owner, survey ID 3848)
	Walking	Where to walk a dog	"*Local walking recommendations*." (Current owner, survey ID 1061)<br>"*About walking areas that are local to goto [sic.] with my dog.*" (Potential owner, survey ID 10305)
	Local	Local amenities	"*I've researched vets in the local area too*." (Potential owner, survey ID 660)
How to source a dog	How to source a dog	Where to get a dog from	"*Where to get a dog rescue/buy.*" (Current owner, survey ID 1995)
		Comparison of sources	"*I looked at blogs about the pros and cons of adopting a dog versus buying a puppy from a breeder. I also read about adopting an older dog as opposed to getting a puppy*." (Potential owner, survey ID 1134)
	Breeder	How to source a dog from a breeder	"*Re-read Kennel Club advice about buying a puppy. Got list of approved breeders from The Schnauzer Club. Phoned a couple of breeders I had heard of that were on the list. Spoke with our prefered [sic.] breeder a few times before visiting her and her dogs*." (Current owner, survey ID 8315)
		How to find a reputable breeder<br>- Avoidance of breeding that has a negative impact on dog welfare	"*Sourcing a good breeder*." (Current owner, survey ID 1764)<br>"*I have been looking into reputable breeders*." (Potential owner, survey ID P1669)<br>*"Where to get a dog from that doesn't involve puppy farming, cruelty and is ethical."* (Potential owner, survey ID 4200)
		Registered, accredited or assured	"*Where to find recognised & registered breeders*." (Current owner, survey ID 1990)<br>"*Checked breeder was KC accredited. Checked with a person I had my previous dog from that she knew the breeder and she was genuine and careful in her breeding.*" (Current owner, survey ID 7930
		Questions to ask	*"What questions to ask of a breeder when looking for a pedigree dog."* (Current owner, survey ID 3012)<br>*"What questions to ask when looking for puppies."* (Potential owner, survey ID 2419)<br>*"Advice on what questions to ask (can parents be seen, background, temperament, health etc)."* (Potential owner, survey ID 7888)

	Rescue or rehome	How to adopt a dog	*"Read all info on any rescue websites on what was needed to adopt a dog."* (Current owner, survey ID 3690)<br>*"Researched online about where to find rescue dogs."* (Current owner, survey ID 6185)
		The rehoming process	*"Which rescue centres were the best and how the process worked.*" (Current owner, survey ID 1169)<br>*"Process of adopting a dog from a rescue centre."* (Potential owner, survey ID 2215)
		Oversees<br><br>- Special support	"*[Dog's name] came from a Spanish rescue so I did research on Spanish dogs, illnesses as well as the rescue centre*." (Current owner, survey ID 1998)<br>"*What different support a rescue from another country might need*." (Potential owner, survey ID 1969)
		Availability of dogs	"*What dogs were available."* (Current owner, survey ID 6849)<br>"*Availability of suitable dogs from local rescue centres*." (Potential owner, survey ID 658)
